# Are customers happy with robot service? Investigating satisfaction with robot service restaurants during the COVID-19 pandemic

**DOI:** 10.1016/j.heliyon.2022.e08986

**Published:** 2022-02-17

**Authors:** Osman El-Said, Sara Al Hajri

**Affiliations:** a Department of Logistics, Tourism, and Service Management, German University of Technology (GUtech), Oman; b Department of Hotel Management, Faculty of Tourism and Hotels, Alexandria University, Egypt

**Keywords:** Autonomous services, Clients, Covid-19, Customers, Experience, Pandemic, Restaurants in Muscat, Robots, Satisfaction

## Abstract

The COVID-19 virus has led to a rapid increase in demand for robot service restaurants. However, given the novelty of both, little is known regarding the impact of this technology on consumer attitudes in the time of the pandemic. Using a sample of customers with dining experiences in Muscat-based robot service restaurants, the current study investigates the factors that affect experience satisfaction and experience extension. The results demonstrate that perceived usefulness, speed of service, and experience novelty directly influence experience satisfaction, while perceived enjoyment and experience satisfaction directly influence experience extension. Moreover, novelty seeking moderates the effects of experience novelty and perceived enjoyment on experience satisfaction, as well as the impact of perceived enjoyment on experience extension. Lastly, perceived risk reduction of infection and trust are found to moderate the impact of experience satisfaction on experience extension. Theoretical and practical implications are offered.

## 1 Introduction

In the last few years, as the technology has become ever more feasible (Jang and Lee, 2020), the subject of robot service has gained increasing attention from both academics and industry practitioners. In the hospitality industry especially, the potential of replacing humans with robots in front line service positions has produced convincing arguments from proponents as well as opponents (Kim et al., 2021). Before the onset of the COVID-19 pandemic, most researchers concluded that there was a general preference for human service. While technological advancements had meant that robots could indeed perform the same basic functions as humans, compelling evidence suggested that they were, so far, unable to recreate important elements of the hospitality experience. Specifically, robots could not mimic the personalized service (Ariffin, 2013), human touch (Kattara and El-Said, 2013), service quality (Choi et al., 2020), sincere interactions (Shin and Jeong, 2020), and experience enrichment (Chan and Tung, 2019), that humans brought into their service delivery.

With the arrival of the pandemic, however, preferences quickly changed (Kim et al., 2021; Wang and Wang, 2021). All high contact service industries including restaurants witnessed a severe decline in patronage (Al-Mughairi et al., 2021; Hou et al., 2021; Lee et al., 2021; Wan et al., 2020; Salem et al., 2021; García-Gómez et al., 2021). Fear of viral infection prompted consumers to seek out restaurants where there was less exposure to other humans (Wan et al., 2020), and the demand for establishments with robot services surged (Wang and Wang, 2021). Some commentators (e.g., Kim et al., 2021; Wang and Wang, 2021) have suggested that increased public awareness of global health risks has resulted in a permanent paradigm shift for the hospitality industry. That is to say, the danger of the pandemic has become so engraved in the public consciousness, that people are likely to maintain their adopted cautious behaviors long after the virus has dissipated (Kim et al., 2021). Consequently, as Matthews (2020) states, it should be expected that more and more restaurants will embrace robot services to meet the technology's growing popularity.

The recency of the technology has meant that, so far, very few scientific studies have addressed the subject of customer satisfaction with, or acceptance of, robot service in restaurants (e.g., Lee et al., 2018; Park, 2020; Seo and Lee, 2021). Of these, an even fewer number have investigated the post-experience behavior of customers (e.g., Hwang et al., 2020; Jang and Lee, 2020). Moreover, due to the recency of COVID-19, few empirical studies have been conducted to explore customer satisfaction, or experience extension intention, with robot service restaurants in the context of the pandemic. Therefore, to support the long-term commercial viability of the hospitality industry, there is a need to study the emerging trend of robot service adoption in a time of global health risks. Particularly, the findings of previous researchers urgently need to be confirmed and extended upon, to build a body of literature capable of guiding practice, both during and after the COVID-19 pandemic.

To address this need, the current study seeks to answer the questions “In the time of the COVID-19 pandemic, what are the factors that affect customer satisfaction with robot service restaurants?” and “In the time of the COVID-19 pandemic, what are the factors that influence customer intention to revisit or recommend robot service restaurants?“. Three objectives have been adopted to answer the research questions. First, to build a comprehensive model that will measure satisfaction with robot service restaurants during the COVID-19 pandemic. Second, to widen the model to measure experience extension intentions. Third, to empirically test the model on a sample of customers with recent experiences in Muscat-based robot service restaurants.

The city of Muscat, in the Sultanate of Oman, provides a useful opportunity for this study. Previous research on robot service restaurants has, so far, covered South Korea (Hwang et al., 2020; Seo and Lee, 2021; Jang and Lee, 2020), Taiwan (Lee et al., 2018), the United States of America (Park, 2020), and China (Qiu et al., 2020). The Arabian gulf region, however, has remained completely uninvestigated. As such, conducting the study in this part of the world will provide valuable insights into the generalizability of previous research. Furthermore, though the concept is still fresh throughout the region, Muscat already features two robot service restaurants. Therefore, with customers from the two different restaurants, a sizeable sample can be extracted, allowing the proposed model to be tested with greater accuracy.

Ultimately, the findings of the current study will have theoretical and practical utility. From a theoretical perspective, a valuable addition will be made to the emerging, but sorely limited, body of knowledge regarding customer satisfaction and experience extension behavior in robot service restaurants. Furthermore, a pioneering contribution will be made regarding reactions to robot service in restaurants during the COVID-19 pandemic, providing much needed understanding of consumer attitudes towards the technology during a global health crisis. From a practical perspective, the results of the current study will offer restaurant managers guidance that, if applied, will improve business performance in a period of particular economic sensitivity. From the recommendations contained within this study, robot service restaurants will be able to meet consumer concerns and improve customer attraction, allowing them to overcome the negative effects of the COVID-19 virus, as well as any future pandemics.

## 2 Literature review

### 2.1 Theoretical background

First, introduced by Davis (1985), the technology acceptance model (TAM) predicts a person's adoption of new technologies using three factors, namely perceived ease of use, perceived usefulness, and perceived enjoyment. In previous research on the public's acceptance of robots, these three factors have proven particularly effective (e.g., De Graaf, 2016). In hospitality contexts, however, researchers have suggested that additional factors may be needed to fully explain the adoption of robot technology (Go et al., 2020). For example, the speedier service that robots offer could impress customers (Lee, 2011). Similarly, the novelty of the robot service experience itself could have a positive impact on customer satisfaction (Bello and Etzel, 1985; Duman and Mattila, 2005).

**Figure 1:**
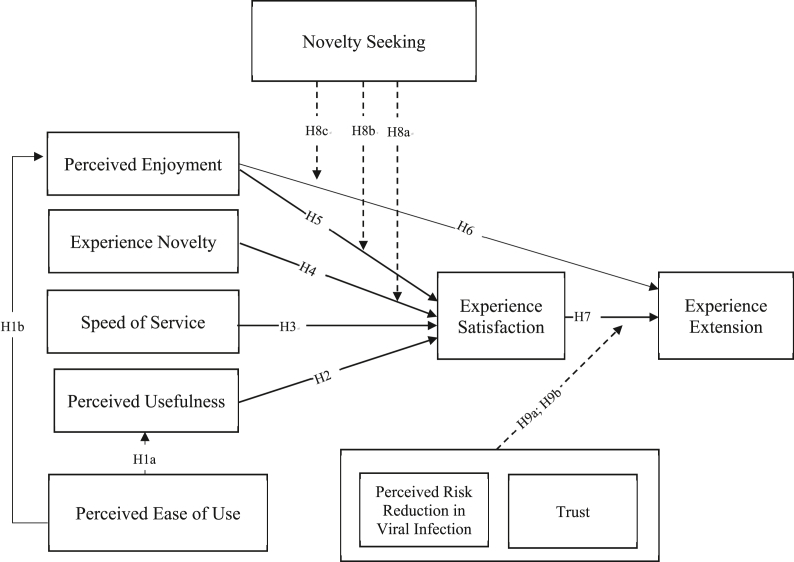
Theoretical model of the current study.

For our study, we have adopted and expanded on the TAM to measure the experience satisfaction (ES) and experience extension (EE) of robot service restaurant (RSR) customers. We have maintained the three original factors of perceived ease of use (PEOU), perceived usefulness (PU), and perceived enjoyment (ENJ), and added experience novelty (EN) and speed of service (SOS) as independent variables. We have also included three moderating variables. Respectively, these are novelty seeking (NS), perceived risk reduction of viral infection (PRR), and trust (TRST). Figure 1 depicts the theoretical model of the study.

### 2.2 Hypotheses development

#### 2.2.1 Effect of perceived ease of use on perceived enjoyment and usefulness

Davis (1989, p. 320) defines perceived ease of use as the “the degree to which a person believes that using a particular technology would be free of effort”. He further notes that the factor should be operationalized as a predictor of perceived usefulness rather than a predictor of attitude towards a technology. Previous research in the context of robot service has validated this statement (e.g., Park and Pobil, 2013; Lee et al., 2018). For example, exploring customer attitudes towards robot service in Korean restaurants, Seo and Lee (2021) demonstrated the strong effect of perceived ease of use on perceived usefulness. The impact of perceived ease of use on perceived enjoyment has been confirmed in several service sector contexts as well. For example, in their investigation of visitor attitudes towards virtual tours for heritage sites, El-Said and Aziz (2021) found that perceived ease of use had a positive impact on perceived enjoyment. Likewise, Rodrigues et al. (2016) determined that perceived ease of use and perceived enjoyment were interrelated in their study of bank customer intentions to adopt gamification software. In much the same way, in the current study, we assume that perceived ease of use will have a positive influence on perceived usefulness and perceived enjoyment for customers of Muscat-based robot service restaurants.

Hypothesis 1a Perceived ease of use has a positive effect on perceived usefulness.

Hypothesis 1b Perceived ease of use has a positive effect on perceived enjoyment.

#### 2.2.2 Determinants of experience satisfaction (perceived usefulness, speed of service, experience novelty and perceived enjoyment)

Perceived usefulness refers to the extent to which individuals believe that implementing a specific technological invention would ultimately improve their work performance (Davis, 1985). Previous studies focusing on robot service in the hospitality industry have found that perceived usefulness bears a strong influence on customer evaluations of the service experience. In their exploration of robots and AI in hotels, Tavitiyaman et al. (2020) found that the accuracy of information, efficiency of information, and multi-lingual functions positively influenced adoption of the technologies. Similarly, El-Said and Al Tall (2020) found that customers' perceptions of the good performance of self-service kiosks in fast food restaurants has a positive impact on their perception of value which in turn affects their intention to re-use the kiosks. For restaurants, robot service possesses many potential advantages. In particular, these include improvements to performance, efficiency, and productivity (Park and Pobil, 2013). In this study, we assume that the perceived usefulness of robot service, as capable of performing tasks accurately and efficiently, will have a positive effect on experience satisfaction for patrons in Muscat-based robot service restaurants.

Hypothesis 2 Perceived usefulness has a positive effect on experience satisfaction.

In general, robots perform tasks faster than humans. In restaurants, this means that the waiting time for orders can be reduced considerably, potentially leading to improved experience satisfaction (Wirtz et al., 2018). The link between quicker service and experience satisfaction in restaurants has previously been identified by Furtado et al. (2020) who explored customer attitudes towards online reservations and online payments. In addition, it should be appreciated that, in restaurants with robot service, the efforts of human staff can be diverted to other, more complex, tasks. In their Turkish study, Vatan and Dogan (2021) reveal how the efficient distribution of tasks, between robots and humans, reduces service time and improves overall efficiency. In this study, we assume that the quicker service offered by robot service will have a positive effect on experience satisfaction for customers of Muscat-based robot service restaurants.

Hypothesis 3 Speed of service has a positive effect on experience satisfaction.

Customer feelings of having tried something new and different is understood as experience novelty (Crompton, 1979). Other synonyms for novelty include curiosity, adventure, new, and different (Crompton, 1979). As noted by previous researchers (e. g., Hwang et al., 2020), experience novelty is one of the main motivators for visiting new places and trying new technologies. Regarding the latter, customers who seek novel experiences tend to have positive perceptions after trying new technologies (Domina et al., 2012). In hospitality contexts, the introduction of robot service is associated with enhanced guest experiences, brought on by the novelty of the technology (Qiu et al., 2020). In the current study, we assume that experience satisfaction will be positively influenced by experience novelty for patrons of Muscat-based robot service restaurants.

Hypothesis 4 Experience novelty has a positive effect on experience satisfaction.

The extent to which a specific technology is considered to be enjoyable, regardless of its performance, is understood as perceived enjoyment (Davis et al., 1992). Across multiple service sectors, perceived enjoyment has proven to be a significant predictor of customer acceptance of new technologies. In their study, Hwang et al. (2019) found that perceived enjoyment affected consumer attitude towards drone food delivery services. Similarly, Hwang et al. (2020) found that perceived enjoyment, or hedonically motivated consumer innovativeness in their study, had a significant impact on customer attitude towards robot service. In our study, we assume that perceived enjoyment has a positive effect on experience satisfaction for customers of Muscat-based robot service restaurants.

Hypothesis 5 Perceived enjoyment has a positive effect on experience satisfaction.

#### 2.2.3 Factors affecting experience extension

Experience extension refers to “visitors’ efforts to share the perceived affective and/or cognitive benefits from the experience, often by telling their friends” (Dong and Siu, 2013, p. 544). Previous research reveals how customers are more likely to share and extend their experience if their expectations are met (Dong and Siu, 2013; El-Said et al., 2021). Similarly, the more enjoyable the experience, the more likely a person is to share and extend it with others. This is supported by the work of Mihić and Kursan Milaković, 2017, who demonstrated the positive impact of enjoyable shopping experiences on word-of-mouth (WOM) recommendations among Croatian customers. Such findings are reinforced by Rouibah et al. (2021) who similarly revealed the positive influence of enjoyable e-commerce experiences on electronic WOM. As such, we assume that both experience satisfaction and perceived enjoyment will have a positive effect on experience extension.

Hypothesis 7 Experience satisfaction has a positive effect on experience extension.

Hypothesis 6 Perceived enjoyment has a positive effect on experience extension.

#### 2.2.4 The moderating effects of novelty seeking, trust, and perceived risk reduction

In the current study we define the concept of novelty seeking using the personality approach. Accordingly, there are two main forms of novelty seeking: *inherent* and *actualized* (Hirschman, 1980). “Inherent novelty seeking is viewed as the desire of the individual to seek out novel stimuli; actualized novelty seeking represents the actual behavior by the individual to acquire novel stimuli” (Hirschman, 1980, p. 284). Therefore, novelty-seeking is based on the need to shift from common to unique experiences. This is exemplified by the work of Keaveney (1995). In the former, it is shown that consumers sometimes switch products, not because they are dissatisfied with their current product, but simply to fulfill their need for novelty. In the latter, it is revealed that the search for the “strangeness” and “novelty” of further landscapes, cultures and lifeways, is what motives tourists to leave their usual environment.

Preceding researchers have often identified novelty seeking as a moderating factor rather than an independent one. For example, Chang et al. (2006) found that customers with high levels of novelty seeking tended to evaluate new experience more favorably than customers with low levels of novelty seeking. Assaker and Hallak (2013) found that, in Mediterranean destinations, the level of novelty seeking held by European visitors moderated the relationship between destination image, satisfaction, and re-visit intention. Similarly, exploring the behavior of Chinese tourists at restaurants in Macau, Ji et al. (2016) found that novelty seeking moderated the relationship between willingness to try ethnic food and experience satisfaction. Unfortunately, literature on the moderating effect of novelty seeking in the context of robot service is lacking. Therefore, based on studies in other contexts, we assume that novelty seeking will moderate the relationship between experience satisfaction and experience novelty, as well as experience satisfaction and perceived enjoyment. Moreover, we assume that the impact of perceived enjoyment on experience extension will be moderated by novelty seeking as well.

Hypothesis 8a Novelty seeking moderates the relationship between experience novelty and experience satisfaction.

Hypothesis 8b Novelty seeking moderates the relationship between perceived enjoyment and experience satisfaction.

Hypothesis 8c Novelty seeking moderates the relationship between perceived enjoyment and experience extension.

In their research on robot service, Tussyadiah et al. (2020) define trust as the judgment a person makes about the technology's ability to perform according to their expectations. In their work they also expose how trust has a direct impact on the adoption of and satisfaction with new technologies. Similarly, Park's (2020) work demonstrated the positive impact of trust on consumer intention to dine in robot service restaurants. Unfortunately, within this context, there is a lack of studies investigating the moderating effect of trust on the relationship between experience satisfaction and experience extension. However, some insight can be withdrawn from exploring trust in the context of media and politics. Ardèvol-Abreu et al. (2018) found that trust of traditional media moderated the impact of user generated news on online political participation. In this vein, for our study, we assume that trust will moderate the impact of experience satisfaction on experience extension for customers of Muscat-based robot service restaurants.

Hypothesis 9a Trust moderates the relationship between experience satisfaction and experience extension.

Studies have shown that, in the wake of the COVID-19 pandemic, consumer attitudes towards technology-based solutions, like robot service, have become more favorable (Kim et al., 2021; Zeng et al., 2020). Concerns of viral exposure have prompted a great many people into seeking out human-less alternatives for everyday activities, like restaurant dining (Lee et al., 2021). In their experimental study of hotel stays during the pandemic, Kim et al. (2021) found that guest attitudes were more positive in hotels staffed by robots than in hotels staffed by humans. Likewise, Hou et al. (2021) identified a preference for robot, rather than human, services in highly crowded touristic destinations. In their study, as robot service reduced the need for interaction with other people, customers felt less at risk of exposure to the virus. In much the same way, for the current study, we assume that the perceived risk reduction of viral infection will moderate the impact of experience satisfaction on experience extension for patrons of Muscat-based robot service restaurants.

Hypothesis 9b Perceived risk reduction of viral infection moderates the relationship between experience satisfaction and experience extension.

## 3 Methodology

### 3.1 Research context

**Figure 2:**
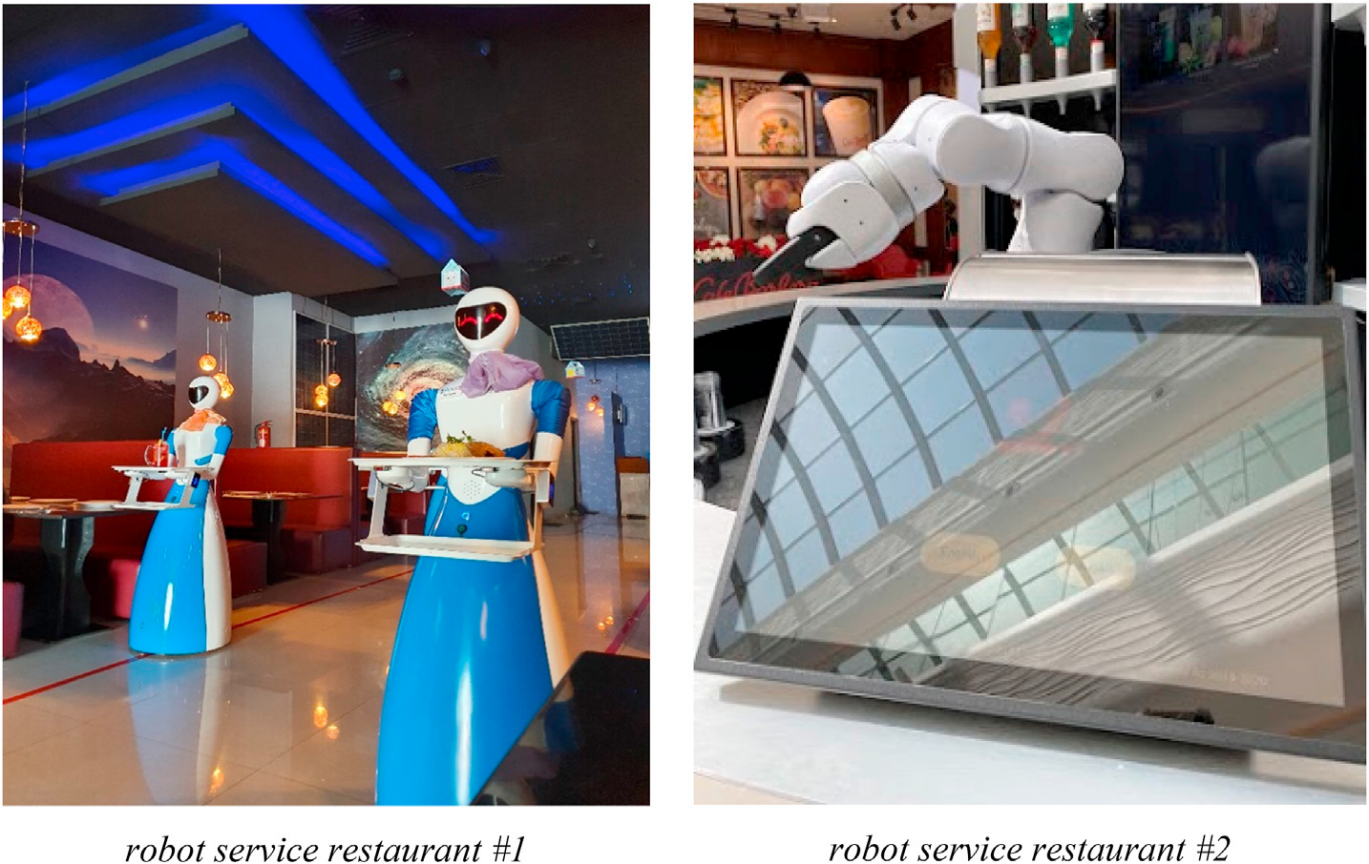
Images for the two robot service restaurants in Muscat.

Data was collected from customers within dining experiences at one of two robot service restaurants located in Muscat during the Covid-19 pandemic. The details of these two robot service restaurants, which we have labelled Restaurant #1 and Restaurant #2, are as follows. Restaurant #1 is a stand-alone establishment located in the commercial zone near the city center with a lively restaurant scene. In this restaurant, robot service is used to compliment human service. Robots deliver food items to tables, while human staff take orders and payments. Restaurant #2 is a coffee shop that is located in a large shopping mall and serves different beverages. In this coffee shop, robot service completely replaces human staff. A robot takes orders, payments, and delivers beverage items. Images of the two robot service restaurants are exhibited in Figure 2. Despite the different nature of the service in the two restaurants, the homogeneity of the sample can be confirmed by a number of factors. The study only concentrated on the robot service component of the greater restaurant experience, and all the survey questions were geared towards the direct interactions that customers had with the robots. Other aspects of the restaurant experience, such as food quality and decoration, were not within the scope of this study, and, therefore, the differences between the two restaurants were negligible. However, to verify the normal distribution of the data, the data was tested by calculating the values for both kurtosis and skewness, which demonstrated that the data was normally distributed.

### 3.2 Data collection procedure

Due to COVID-19 related restrictions, such as the city-wide lockdown, it was not possible to distribute the survey physically. Therefore, the data collection was carried out using an online survey. Convenience sampling was used, and the sampling frame included customers who had visited one of the robot service restaurants after the start of the COVID-19 pandemic, and still remembered the experience. A link to the survey was posted on different social media platforms, such as Twitter, Facebook, Instagram, Snapchat, and WhatsApp, to ensure that data was collected from a diverse demographic group. Before collecting the data, all the ethical considerations mandated by the Research Ethics Committee at the German University of Technology in Oman were followed, and required ethical approvals were obtained. The study took into account the following ethical considerations. Respondents were informed that their participation in the study was voluntary, all data would be used for research purposes only, and that their data would be treated strictly confidential. Before starting the survey, respondents were briefed about the purpose and context of the study. Specifically, of the two robot service restaurants in question, and the COVID-19 virus. Respondents were informed in the introduction that they should participate in the survey only if they had dined in one of the two robot service restaurants. Furthermore, an eligibility question asked the respondents if they had visited either of the two relevant robot service restaurants since the start of the COVID-19 pandemic. Pictures of the two robot service restaurants in question were displayed next to this question to avoid any confusion. Participants who answered “Yes, I have tried at least one of them,” were eligible to participate in the following sections of the survey. Those who answered “No, I have not tried any robot service restaurants during COVID-19” were not eligible to complete the survey. Data was collected from the beginning of March until the end of May 2021 and a total of 637 surveys were collected, of which 191 were incomplete and subsequently eliminated. Therefore, there were 446 completed and useable surveys for the data analysis.

### 3.3 Research instrument

The survey was designed and structured to determine experience satisfaction with different characteristics of the robot service restaurants. The survey was composed on an introduction and three sections. In the introduction, the purpose of the research was stated, followed by a brief description of the questions in the following three sections. The first section focused on the demographic attributes of the respondents. The second section consisted of five closed-ended multiple choice questions.

The third section of the survey included questions that captured the latent variables of the study. The factors of perceived usefulness, perceived ease of use and perceived enjoyment were measured using four items each drawn from previous TAM models (e.g., Davis, 1989; Davis et al., 1992) and adapted in subsequent research concentrating on service robots (e.g., Lu et al., 2019; Hwang et al., 2020). speed of service was measured using three items adapted from the work of Lee (2011) and Sheu et al. (2003). Trust was measured using four items derived from the work of Mcknight et al. (2011). Perceived risk reduction of viral infection was measured using three items adapted from Terpstra and Lindell (2013) and Wan et al. (2020). Experience novelty was measured using five items derived from Bello and Etzel (1985) and Duman and Mattila (2005). Novelty-seeking was measured using seven items adapted from Lee and Crompton (1992) and Jang and Feng (2007). Experience satisfaction was measured using a three-item scale adapted from Tussyadiah et al. (2020) and Dong and Siu (2013). Finally, experience extension was measured using four items derived from the work of Dong and Siu (2013). The responses of this section were measured on a Likert scale consisting of five options that ranged from "strongly disagree" to "strongly agree".

### 3.4 Sample characteristics

Table 1 below displays the respondent demographics. The final sample had 213 males (47.8%) and 233 females (52.2%). Regarding age, 39.5% were between 18 and 30, 26.9%, were between 31 and 45, 13.7% were between 46 and 60, 12.6% were below 18, and 7.4% were 61 years and above. Concerning marital status, 48% were single and 46.9% were married. Most of the respondents, 83.2%, found the restaurants family-friendly, while the remaining 16.8% did not. For visit frequency, 47.3% of the respondents had visited a robot service restaurant visited once, 32.7% had visited twice, and 20% had visited more than two times. In terms of company, 45.5% of the respondents went alone to the restaurant robot service restaurants, 37.2% went with family or relatives, and 17.3% went with friends. Most of the respondents, 76.7%, were pleased with the food offered at the robot service restaurants, and 23.3% were not pleased. Regarding general dining habits during the COVID-19 pandemic, 43.8% went out to restaurants often, 26.2% always ate out, and 30% ate out rarely.

**Table 1:** Profile of respondents.

	*Total (N = 446)*	
	*N*	*%*
***Gender***		
Male	213	47.8
Female	233	52.2
***Marital Status***		
Single	214	48.0
Married	209	46.9
Other	23	5.1
***Age***		
*Less than 18*	56	12.6
*18–30*	176	39.5
*31–45*	120	26.9
46–60	61	13.7
61 and above	33	7.4
***Did you find the restaurant/Cafe family-friendly?***		
Yes	371	83.2
No	75	16.8
***How many times did you visit a robot service restaurant/cafe?***		
More than two times	89	20.0
Once	211	47.3
Twice	146	32.7
***With whom did you visit the restaurant/cafe?***		
Alone	203	45.5
With family/relatives	166	37.2
With friends	77	17.3
***Were you pleased with the quality of food/drinks served?***		
No	104	23.3
Yes	342	76.7
***How often do you eat out during the COVID-19 pandemic?***		
Always	116	26.0
Often	195	43.7
Rarely	134	30.0

## 4 Results

### 4.1 Data screening and analysis

The data was analyzed using SPSS version 25, AMOS version 24, and PROCESS Macro for SPSS version 3.2. The data was assessed for any issues affecting the quality of the research findings before starting the analysis process. Namely, the data was tested for common method bias, normality, and multicollinearity. To test for common method bias, Harman's Single Factor test was performed using Principal Axis Factoring as the method of extraction. Results indicated that a single factor, on which all the observed variables were loaded, attributed for less than 40 % of the variance, confirming the absence of common method bias (Harman, 1967). To check for data normality, the values for both kurtosis and skewness were calculated and found to be less than ±1. The deviation confirmed the normal distribution of the data collected (Hair et al., 2010). To check for multicollinearity between the independent variables, Variable Inflation Factor (VIF) values were calculated. At less than 3, the values confirmed the non-existence of multicollinearity (Hair et al., 2010).

### 4.2 Confirmatory factor analysis

A confirmatory factor analysis (CFA) was performed to test the convergent validity, discriminant validity and reliability of the study's constructs. An initial CFA indicated an average fit to the data. After checking the loadings of the observed variables and the cross-loadings among variables from different constructs, PU4 was excluded from the perceived usefulness construct, TRST1 was excluded from the trust construct, EN2 was excluded from the experience novelty construct, and NS4 and NS6 were excluded from the novelty seeking construct. Previous variables were excluded due to poor loading (below. 6) or high cross loading with other variables. A second CFA was performed, and the results indicated that the adjusted model showed a good fit to the data with acceptable fit indices values (χ2 = 939.710, df = 539, p < .001, χ2/df = 1.743, GFI = 0.901, CFI = 0.966, TLI = 0.961, IFI = 967, SRMR = 0.0334, RMSEA = 0.041) (Hair et al., 2010).

Tables 2 and 3 show the reliability and validity of the constructs. All the values of the Cronbach's alphas (α) and composite reliability (CR) for the different constructs were greater than the threshold value of .70, confirming the reliability of all constructs (Hair et al., 2010). Furthermore, all the observed variables had high loadings on the respective latent variables with all factor loadings greater than .70, confirming the convergent validity of the constructs. In addition, the AVE for all the constructs was higher than the cut-off value of 0.50, further confirming the convergent validity of the constructs (Malhotra and Dash, 2011). Finally, discriminant validity was assessed and confirmed in two ways. First, the square root of the AVE for each construct was greater than the inter-construct correlations (Fornell and Larcker, 1981). Second, the Maximum Shared Variance (MSV) for each construct was lower than the Average Variance Extracted (AVE) for the same construct. Therefore, there were no validity concerns for the instruments.

**Table 2:** Reliability and validity.

Latent and observed variables	Loading	Skew	Kurt	α	CR	AVE
**Perceived Usefulness (PU)**				**.931**	**0.931**	**0.819**
PU1: Service robots are useful in Muscat-based restaurants	.891	-.264	-.436			
PU2: Service robots were able perform same functions as employees	.919	-.429	-.130			
PU3: Service robots enhanced my service experience	.904	-.496	-.095			
PU4: Service robots had all functionalities needed to do its job	dropped					
**Perceived Ease of Use (PEOU)**				**.940**	**0.938**	**0.792**
PEOU1: Using the service robot did not require a lot of mental effort	.870	-.505	-.335			
PEOU2: I found the service robot to be easy to use	.930	-.585	-.186			
PEOU3: My interaction with the service robot was understandable	.896	-.690	.024			
PEOU4: It was easy to learn how to use the service robot	.863	-.618	-.226			
**Perceived Enjoyment (ENJ)**				**.897**	**0.892**	**0.674**
PE1: Using the service robot gave me lots of pleasure	.796	-.312	-.755			
PE2: The service robot experience was pleasant	.841	-.349	-.799			
PE3: The technological newness of the restaurant service robot made me happier	.821	-.371	-.618			
PE4: The interaction with the service robots in the restaurant was fun	.826	-.410	-.526			
**Speed of Service (SOS)**				**.774**	**0.776**	**0.536**
SOS1: The robot was efficient in serving my food/drinks on time	.717	-.325	-.842			
SOS2: Enjoying the experience made the time pass quickly and I didn't feel the waiting time	.730	-.288	-.792			
SOS3: I did not wait long till my food/drinks arrived	.749	-.326	-.880			
**Trust (TRST)**				**.792**	**0.850**	**0.654**
TRST1: The robot had the required features to serve food/drinks	.784	-.262	-.945			
TRST2: The robot provided me with the help I needed	dropped					
TRST3: The robot served food/drinks error-free (without mistakes)	.792	-.431	-.895			
TRST4: I trust that the service robots in the restaurants are safe	.849	-.373	-.778			
**Perceived Risk Reduction of Viral Infection (PRR)**				**.835**	**0.812**	**0.591**
PRR1: Service robots are a safe alternative that protect me from catching COVID-19	.764	-.538	-.564			
PRR2: Compared to human employees, service robots can limit the spread of COVID-19 in restaurants	.808	-.462	-.610			
PRR3: I prefer being served by robots instead of human employees during COVID-19	.733	-.446	-.701			
**Experience Novelty (EN)**				**.860**	**0.854**	**0.595**
EN1: Eating at the robot restaurant was a unique experience for me	.772	-.433	-.850			
EN2: I think most people would describe this experience as different	dropped					
EN3: Being served by a robot was a new experience for me	.767	-.566	-.510			
EN4: The service at this restaurant is different from the service at other restaurants	.739	-.356	-.891			
EN5: After eating at this restaurant, I believe that I did something new and different	.805	-.346	-.866			
**Experience Satisfaction (ES)**				**.837**	**0.874**	**0.699**
ES1: My overall experience was satisfactory	.777	-.494	.434			
ES2: My overall experience was memorable	.848	-.538	.182			
ES3: My overall experience was enjoyable	.880	-.602	.194			
**Experience Extension (EE)**				**.907**	**0.898**	**0.688**
EE1: I would recommend this robot service restaurant to someone	.750	-.551	-.159			
EE2: I would encourage friends and relatives to visit this robot service restaurant	.876	-.439	-.286			
EE3: I would share this experience with my friends and relatives	.799	-.584	.048			
EE4: I will visit this robot service restaurant again	.886	-.427	-.233			
**Novelty Seeking (NS)**				**.915**	**0.920**	**0.697**
NS1: Change of environment allows me to experience something new	.792	-.392	-.481			
NS2: I like to visit new places	.844	-.499	-.508			
NS3: I like to learn about different cultures from my own	.867	-.438	-.732			
NS4: I like trying new food	dropped					
NS5: I like Experiencing new and different things	.839	-.460	-.597			
NS6: I Feel an urge to explore the unknown	dropped					
NS7: I like visiting a place I can talk about when I get home	.832	-.411	-.848

**Table 3:** Inter construct correlations and square roots of AVE.

	MSV	EE	PU	PEOU	PE	SOS	TRST	PRR	EN	ES	NS
EE	0.461	**0.830**									
PU	0.333	0.412	**0.905**								
PEOU	0.483	0.483	0.577	**0.890**		Read Caveats and Assumptions below, and then click me.					
ENJ	0.517	0.592	0.547	0.661	**0.821**						
SOS	0.508	0.679	0.569	0.648	0.713	**0.732**					
TRST	0.564	0.596	0.519	0.695	0.666	0.678	**0.809**				
PRR	0.564	0.607	0.575	0.678	0.649	0.671	0.751	**0.769**			
EN	0.555	0.584	0.385	0.655	0.719	0.640	0.745	0.684	**0.771**		
ES	0.423	0.650	0.434	0.409	0.400	0.544	0.416	0.534	0.458	**0.836**	
NS	0.288	0.537	0.187	0.367	0.327	0.381	0.428	0.512	0.412	0.409	**0.835**

Note: square root of AVE is shown in the diagonal and in bold.

### 4.3 Structural model and hypothesis testing

After confirming the validity and reliability of the model, the hypotheses of the study were tested by analyzing the path coefficients between the latent variables in the structural model. Results of the structural model revealed a good model fit to the data and all the fit indices were acceptable (χ2 = 622.530, df = 260, p < .001, χ2/df = 2.394, GFI = 0.903, CFI = 0.957, TLI = 0.950, IFI = 957, SRMR = 0.0737, RMSEA = 0.056). As shown in Table 4, both standardized regression weights and p-values were used to test the different hypotheses of the study. The results show that perceived ease of use has a positive and significant impact on perceived usefulness (β = .594, p < 0.001) and perceived enjoyment (β = .680, p < 0.001), supporting hypotheses H1a and H1b. Moreover, the results indicate that perceived usefulness (β = .208, p < 0.001), speed of service (β = .361, p < 0.001), and experience novelty (β = .204, p < 0.01) have a positive and significant impact on experience satisfaction, supporting hypotheses H2, H3 and H4. However, perceived enjoyment has no significant impact on experience satisfaction (β = -.076, p > 0.05), hence H5 was rejected. Finally, both perceived enjoyment (β = .393, p < 0.001) and experience satisfaction (β = .529, p < 0.001) have a positive and significant impact on experience extension, providing support for hypotheses H6 and H7.

**Table 4:** Results of testing the structural model.

Predictor		Dependent Variable	Estimate	t-value	P-value	R^2^	Decision
H1a: PEOU	--->	PU	.594	12.682	.000∗∗∗	.353	Supported
H1b: PEOU	--->	PE	.680	13.610	.000∗∗∗	.462	Supported
H2: PU	--->	ES	.208	3.975	.000∗∗∗	.355	Supported
H3: SOS	--->	ES	.361	4.739	.000∗∗∗		Supported
H4: EN	--->	ES	.204	2.960	.003∗∗		Supported
H5: ENJ	--->	ES	-.076	-1.400	.161		Not Supported
H6: PE	--->	EE	.393	9.008	.000∗∗∗	.543	Supported
H7: ES	--->	EE	.529	11.041	.000∗∗∗		Supported

*Note;* ∗∗∗p < 0.001; ∗∗p < 0.01; ∗p < 0.05.

### 4.4 Testing the moderating effects

**Figure 3:**
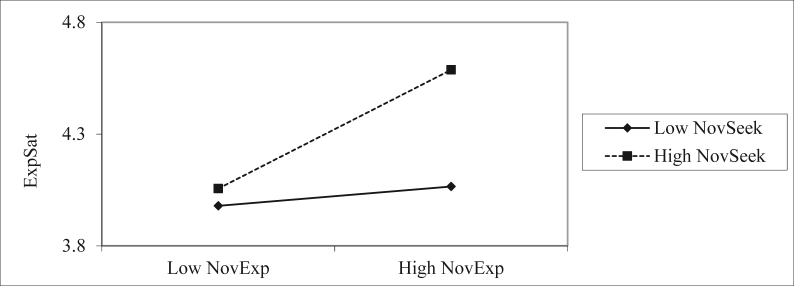
Simple slope between EN and ES at different levels of NS.

**Figure 4:**
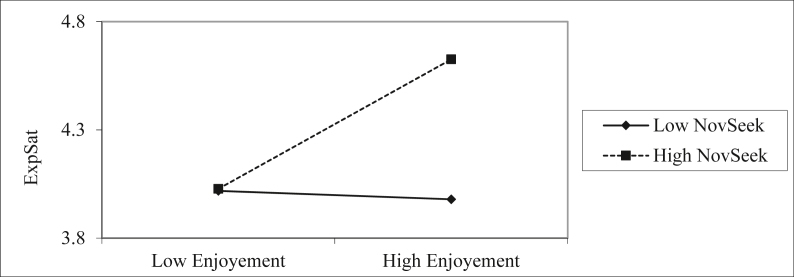
Simple slope between ENJ and ES at different levels of NS.

**Figure 5:**
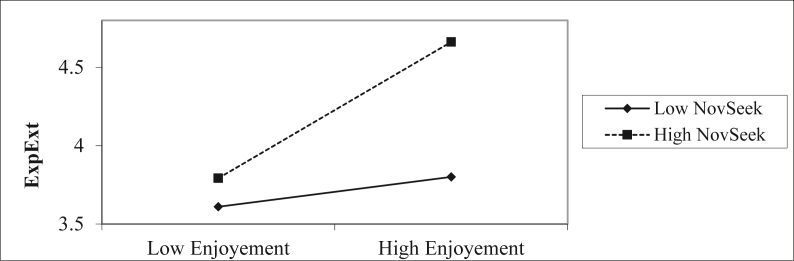
Simple slope between ENJ and EE at different levels of NS.

**Figure 6:**
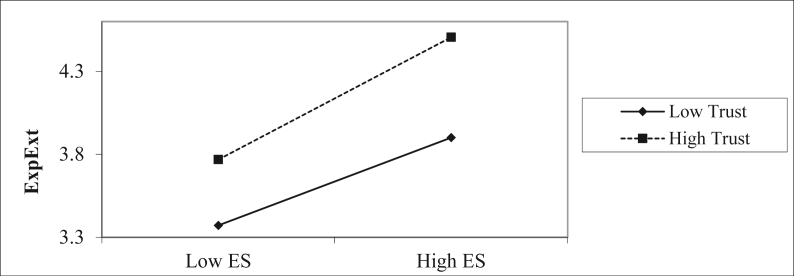
Simple slope between ES and EE at different levels of TRST.

**Figure 7:**
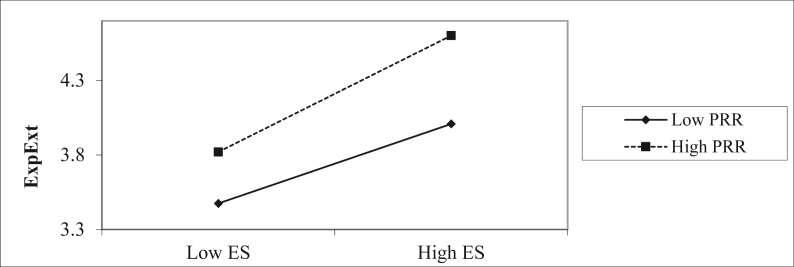
Simple slope between ES and EE at different levels of PRR.

The moderating effects were tested using the Process Macro version 3.2, model 1 (Hayes, 2017). Results of the moderating effects from these tests are reported in Table 5 and plotted in Figures 3, 4, 5, 6, and 7. Model 1 shows that there is a significant positive moderating effect for novelty seeking on the relationship between experience novelty and experience satisfaction (R^2^-chng = .0315, F = 18.37, p < 0.001). Furthermore, the result of the conditional analysis, which is plotted in Figure 3, shows that when novelty seeking is high, the positive impact of experience novelty on experience satisfaction increases (b = .357, t = 7.68, p < .001), and when novelty seeking is low, the positive impact of experience novelty on experience satisfaction significantly decreases (b = .026, t = .479, p > .05). Therefore, Hypothesis 8a is supported. Model 2 shows that there is a significant positive moderating effect for novelty seeking on the relationship between perceived enjoyment and experience satisfaction (R^2^-chng = .0672, F = 40.55, p < 0.001). Additionally, the result of the conditional analysis, which is plotted in Figure 4, shows that when novelty seeking is high, the positive impact of perceived enjoyment on experience satisfaction increases (b = .423, t = 8.49, p < .001), and when novelty seeking is low, the positive impact of perceived enjoyment on experience satisfaction significantly decreases (b = -.061, t = -1.17, p > .05). Therefore, hypothesis 8b is supported. Model 3 shows that there is a significant positive moderating effect for novelty seeking on the relationship between perceived enjoyment and experience extension (R^2^-chng = .0528, F = 41.67, p < 0.001). Moreover, the conditional analysis results, which are plotted in Figure 5, show that perceived enjoyment has a stronger positive effect on experience extension for customers with higher levels of novelty seeking (b = .595, t = 11.95, p < .001) than those with lower levels of novelty seeking (b = .078, t = 1.42, p > .05). Therefore, hypothesis 8c is supported.

**Table 5:** Moderation analysis.

	Coefficient	SE	t	p		LLCI	ULCI
***Model 1: Impact of EN on ES at different levels NS***							
*Constant*	5.653	0.768	7.359		0.000	4.144	7.163
*EN*	-0.620	0.197	-3.139		0.002	-1.008	-0.232
*NS*	-0.534	0.182	-2.928		0.004	-0.892	-0.176
*EN X NS*	0.196	0.046	4.287		0.000	0.106	0.285
*R2/Sig.*	*.4915/.000*						
*R2 change/Sig.*	*.0315/.000*						

***Model 2: Impact of ENJ on ES at different levels NS***							
*Constant*	7.002	0.740	9.462		0.000	5.548	8.457
*PE*	-1.005	0.192	-5.235		0.000	-1.382	-0.628
*NS*	-0.849	0.177	-4.801		0.000	-1.196	-0.501
*PE X NS*	0.286	0.045	6.368		0.000	0.198	0.374
*R2/Sig.*	*.2680/.000*						
*R2 change/Sig.*	*.0672/.000*						

***Model 3: Impact of ENJ on EE at different levels of NS***							
*Constant*	5.935	0.779	7.614		0.000	4.403	7.466
*PE*	-0.929	0.202	-4.596		0.000	-1.327	-0.532
*NS*	-0.780	0.186	-4.191		0.000	-1.146	-0.414
*PE x NS*	0.305	0.047	6.455		0.000	0.212	0.398
*R2/Sig.*	*.6636/.000*						
*R2 change/Sig.*	*.0528/.0007*						

***Model 4: Impact of ES on EE at different levels of TRST***							
*Constant*	2.171	0.630	3.445		0.001	0.932	3.410
*ES*	0.154	0.153	1.010		0.313	-0.146	0.454
*TRST*	-0.126	0.173	-0.730		0.466	-0.466	0.214
*ES X TRST*	0.105	0.041	2.584		0.010	0.025	0.185
*R2/Sig.*	*.6825/.000*						
*R2 change/Sig.*	*.0081.0101*						

***Model 5: Impact of ES on EE at different levels of PRR***							
*Constant*	2.475	0.606	4.086		0.000	1.285	3.665
*ES*	0.127	0.151	0.843		0.400	-0.169	0.423
*PRR*	-0.208	0.168	-1.243		0.215	-0.537	0.121
*ES X PRR*	0.111	0.040	2.795		0.005	0.033	0.190
*R2/Sig.*	*.6573/.000*						
*R2 change/Sig.*	*.0100/.0054*

Model 4 shows that there is a significant positive moderating effect for trust on the relationship between experience satisfaction and experience extension (R^2^-chng = .0081, F = 6.67, p < 0.05). Additionally, the conditional analysis results, plotted in Figure 6, show that the positive impact of experience satisfaction on experience extension increases when trust is high (b = .678, t = 9.30, p < .001), and the positive impact of experience satisfaction on experience extension decreases when trust is low (b = .468, t = 9.10, p < .001). Therefore, Hypothesis 9a is supported. Model 5 shows that there is a significant positive moderating effect for perceived risk reduction of viral infection on the relationship between experience satisfaction and experience extension (R^2^-chng = .0100, F = 7.80, p < 0.01). Furthermore, conditional analysis plotted in Figure 7, show that experience satisfaction has a stronger effect on experience extension for customers with higher levels perceived risk reduction of viral infection (b = .683, t = 9.12, p < .001) than for those who have lower levels (b = .460, t = 8.55, p < .001). Therefore, hypothesis 9b is supported.

## 5 Discussion

The positive and significant impact of perceived ease of use on perceived usefulness and perceived enjoyment is an expected finding given its basis in the TAM. Regarding the effect on perceived usefulness, it should be appreciated that the easier the technology is to use, the more useful it is for the user. This result is consistent with the findings of previous researchers (Park and Pobil, 2013; Lee et al., 2018). For example, based on a sample of general managers and deputy managers in more than 100 restaurants in Taiwan, Lee et al. (2018) found that perceived ease of use significantly increased perceived usefulness of restaurant service robots. Interpreting the impact on perceived enjoyment, it is understood that the easier the technology is to use, the more enjoyable it is for the user. This finding is also aligned with the work of previous researchers (El-Said and Aziz, 2021). The strong positive impact of perceived usefulness on experience satisfaction indicates that, to a high degree, acceptance of robot service is related to the technology's ability to perform as expected. Essentially, this translates into the robot's ability to take and serve orders accurately. It stands, therefore, that in restaurants with poorly performing robots, where orders are inaccurately or poorly delivered, there will be non-acceptance of the technology from customers. Accordingly, the work of Tavitiyaman et al. (2020) is reinforced. However, in a surprising outcome, perceived enjoyment does not exhibit any significant impact on experience satisfaction. A possible explanation is that the relationship is affected by other factors, as some of the qualitative comments suggested. As one respondent stated, “what made the experience at this restaurant less enjoyable is the quality of food more than the service, since it's a restaurant the food quality is very important, and not only the experience of robots”. Therefore, the findings of the current study disagree with those of preceding researchers (Hwang et al., 2019).

Speed of service displayed the strongest impact on experience satisfaction. This indicates that the time required for robots to take and serve orders was the most important factor for customers. Accordingly, the faster the service, the more satisfied customers will be with robot service, and vice versa. Therefore, the findings of Furtado et al. (2020) are corroborated. In addition, respondent satisfaction with the quick service could indicate the improved productivity and efficiency that robot service brought into the restaurants, allowing human-staff to concentrate their attentions on other elements of the experience, as mentioned by Vatan, and Dogan (2021). The general rarity of robot service explains the positive and significant impact of experience novelty on experience satisfaction. Given that restaurants with such technologies are both new and few, customers are mainly motivated to eat in these establishments to enjoy different experiences. Therefore, for robot service restaurants, the more uncommon the experience, the more satisfied the customers will be. As such, the findings of the current study agree with those of Qiu et al. (2020).

With respect to the impact on experience extension from both perceived enjoyment and experience satisfaction, it is clear that the more enjoyable and satisfactory the experience, the more likely a customer will seek to extend the experience. Thus, the results reinforce the work of Mihić and Kursan Milaković, 2017 and Rouibah et al. (2021), who similarly found that enjoyable experiences had a positive impact on customer willingness to make recommendations to family and friends. Likewise, the results also support the work of Dong and Siu (2013) and El-Said et al. (2021), such that satisfied customers are more willing to share their experiences with others.

An interesting finding of the study is related to the moderating effects of novelty seeking on the relationships between perceived enjoyment and experience satisfaction, perceived enjoyment and experience extension, and experience novelty and experience satisfaction. In all these relationships, as could be clearly observed from the results of the moderation analysis, novelty seeking was found to moderate the relationship between the independent variables (experience novelty, perceived enjoyment) and the dependent variables (experience satisfaction, experience extension). The impact of the independent variables on the dependent variables were stronger for customers with higher levels of novelty seeking, and noticeable weaker, and even non-existent, for customers with lower levels of novelty seeking. This can be interpreted in the following way. Customers with high levels of novelty seeking visit robot service restaurants to interact and use the robots, to enjoy an experience that is uncommon in other establishments. For these customers, other factors related to the dining experience, such as food quality or restaurant décor, are overshadowed by the excitement of experiencing something new. In contrast, for customers with low levels of novelty seeking, the robot service is not the main motivation for eating at the restaurant. Therefore, they remain critical of all the usual factors of the dining experience. This result is supported by the academic literature, which indicates that customers with high levels of novelty seeking tend to evaluate the new experiences more favorably than customers with low levels of novelty seeking (e.g., Chang et al., 2006; Assaker and Hallak, 2013; Ji et al., 2016).

Finally, perceived risk reduction of viral infection and trust were also proven to have moderating effects on the relation between experience satisfaction and experience extension. With respect to perceived risk reduction in viral infection, it can be concluded that because of the spread Covid-19, people became more concerned about their safety, and they prefer services that incorporate technologies that can minimize the spread of the virus. Therefore, customers who more strongly believe that service robots are a safe alternative to humans and can limit the spread of the virus will be more satisfied with these restaurants and will be eager to pass the experience on to their friends and families who are also looking for a safe place to dine. The previous conclusion is similar with the findings of recent studies that found that People's preferences have shifted towards technology-based restaurants because they represent less risks to public health, and they will recommend those restaurants to others as well (Hou et al., 2021; Kim et al., 2021; Lee et al., 2021). Regarding the moderating effects of trust, we follow a similar reasoning, where customers with higher levels of trust in the service robots and their ability to perform their tasks efficiently will be satisfied with the service and eager to pass their experience to others and vice versa. In a different context, with the use of similar logic, Seo and Lee (2021) found that trust in restaurant service robots has a significant positive impact on customers' satisfaction, which in turn positively affects their intention to visit these restaurants in the future.

## 6 Implications

### 6.1 Theoretical implications

The results of the current study provide a four of novel contributions to theory. First and foremost, this study is the first empirical investigation of customer satisfaction with robot service restaurants during the COVID-19 pandemic. Moreover, it is the first empirical investigation of customer intention to extend the experience, after eating in a robot service restaurant, during the COVID-19 pandemic. Accordingly, valuable understanding of post-experience behavior and attitude have been added to the body of literature, which had, until now, only investigated pre-experience preferences (e.g., Kim et al., 2021; Wan et al., 2020). In doing so, the model used in the current study offers future researchers a foundation for assessing robot-technologies, not only for the COVID-19 pandemic, but for future global health crises as well.

Second, the current study represents the first application of robot service restaurant research in the Arabian gulf region. As such, the generalizability of factors identified in previous work, such as perceived ease of use (Park and Pobil, 2013; Lee et al., 2018; Seo and Lee, 2021) and perceived usefulness (Tavitiyaman et al., 2020), is extended to a region that had previously been neglected. The current study has also introduced new factors, from other hospitality contexts, into the discussion on robot service restaurants. These include experience novelty (Hwang et al., 2020; Qiu et al., 2020), novelty seeking (Assaker and Hallak, 2013; Ji et al., 2016), speed of service (Furtado et al., 2020), and trust (Park, 2020). Therefore, the current study has provided valuable evidence to support the generalizability of previous research, both across geographies and contexts.

Third, the current study has brought new understanding of the perceived enjoyment factor. The findings demonstrate that, for robot service restaurants, perceived enjoyment only exhibits an effect when customer novelty seeking is high. This is a unique finding with far-reaching implications. On the one hand, a contribution is made towards the body of knowledge on the TAM, improving general understanding of technology acceptance. On the other hand, the finding suggests that consumer attitudes towards technology in robot service restaurants, and perhaps in other hospitality settings as well, are more greatly affected by user personality traits and motivations.

Fourth, the current study has demonstrated the moderating effect of trust and perceived risk reduction of viral infection. Regarding the former, this is the first study to introduce trust as a moderating variable. Accordingly, understanding of the applications for this factor have been broadened. Regarding the latter, beyond robot service, this is the first study to examine the influence of customer COVID-19 concerns on post-experience behavior in restaurants. Therefore, the findings of the current study deepen understanding of consumer behavior during the pandemic, and highlight the fact that, it is not just demand for restaurant services that is affected, but how people decide to repeat and recommend their experiences as well.

### 6.2 Practical implications

Whether looking to adopt robot service in a traditional restaurant or trying to improve the performance of an existing robot service restaurant, the findings of the current study offer managers and owners numerous insights. In this study, 69.7% of the respondents admitted to eating at restaurants one or more times per week. Therefore, it should be appreciated that the COVID-19 pandemic has not dissuaded a significant portion of people from eating out. However, as evidenced by the moderating effect of perceived risk reduction of viral infection, though many are still willing to risk going out, they are aware of the danger, and may experience greater stress, worry, and guilt than usual. In turn these affects their decision to dine again at the restaurant, or to recommend it to others. As such, managers who which to improve the performance of their robot service restaurants need to focus on both the recreational needs and hygienic concerns of their customers.

Careful attention should be given to the perceived ease of use of using the robot services. Screens could be installed at each table through which brief introduction and orientation videos are played, informing the customer of how to use the robots. These screens could use touch technology, and orders to robots, and the restaurant, could be conducted directly through these screens. On-screen commands should be straightforward and could use simple point and click functions. Efforts should be made to improve the perceived usefulness and perceived enjoyment of the robots. Regarding the former, the robots must be designed to customize orders and take special requests. For example, the robot service must be able to add extra items, remove specific ingredients, swap items. The robot service should also be able to offer different order sizes and prioritize orders. Moreover, the robot service should have a function for quickly and easily reporting service errors. Concerning perceived enjoyment, the robot service experience should be stimulating and engaging. The use of multimedia can enhance the enjoyment of basic functions, like making and receiving orders. Music could be played, or lights could flicker, when orders are completed or when food arrives at the tables. While waiting for food, guests could play digital games, through their robot, or using other interfaces at their table. Furthermore, guests could customize their own eating space.

Managers should concentrate on improving the experience novelty at their robot service restaurants. The restaurant itself could be uniquely themed to reflect the novelty of using robots, or even use multiple themes in rotation. The food itself could also be used to enhance the novelty of the robot services. This could include creative menu items and unique. In addition, certain activities could highlight the strangeness of the experience. For example, the service robot could take a photo of the customer and send it to the customers telephone, so that they can share it on social media. Also, diners could leave digital notes for the next customers using their table to read. As speed of service exhibits the strongest influence on ES, special attention should be paid to this factor. On the one hand this requires making systems and processes more efficient, and, on the other, highlighting the quick service time. For the former, systems to reduce cooking time, table turnover time, and errors. For the latter, menus could mention the preparation time of each item, and timers could be installed at each table to inform the customer of the wait.

As novelty seeking influences both experience satisfaction and experience extension, robot service restaurant managers should target these customers more intensely than others. Advertisements could be made on particular social media and web platforms that discuss technological innovations and/or uncommon hospitality experiences. Moreover, partnerships could be made with other nearby, novelty-focused, product and service providers. For example, a computer gadget shop could offer customers a discount coupon for the robot service restaurant with every purchase, or a nearby robot service hotel could package their accommodation with an experience at the robot service restaurant.

Customer perceptions are particularly important for revisit and recommendation intentions. Therefore, trust and perceived risk reduction of viral infection need to be addressed appropriately. To improve trust perceptions, robot service restaurant managers should link all of their online distribution channels to suitable rating and review platforms (e.g., Trip Advisor, Trustpilot, etc.). Furthermore, positive scores should be highlighted to customers within the restaurant before their experience begins. For example, when the robot first comes to the guest, reference to the trustworthiness of the service can be incorporated into a greeting, as in “Welcome to … Enjoy our 5-star service, as rated on Trip Advisor!“. For perceived risk reduction of viral infection, managers should ensure that hygiene procedures are satisfactory, and then make sure to communicate these measures to all customers. Regarding the former, there are numerous ways to improve COVID-19 related hygiene in restaurants. Regarding the latter, it is crucial that customers understand the protective measures taken during their dining experience. On arrival at a table, a small video or infographic could be played reviewing all the disinfection procedures used between each group of diners. Furthermore, when the robots take orders, they could give a very brief introduction into how they are cleaned at each trip to and from the kitchen, as well as the hygiene procedures used by the kitchen staff.

## 7 Conclusion and directions for future research

This study set out to achieve three research objectives. A model was successfully built, extended, and empirically tested on a sample of customers with experiences in Muscat-based robot service restaurants. Overall, this study aimed to answer two research questions. Regarding the first question, the factors that affect customer satisfaction with robot service restaurants in the time of the COVID-19 pandemic are as follows. Perceived ease of use has a strong direct impact on both perceived usefulness and perceived enjoyment. In turn perceived usefulness, along with speed of service and experience novelty, have strong direct impacts on experience satisfaction. Moreover, the effect of experience novelty on experience satisfaction is moderated by the novelty seeking tendency of customers. However, perceived enjoyment does not influence experience satisfaction, except when customers have high levels of novelty seeking. Regarding the second question, the factors that affect customer intention to revisit or recommend robot service restaurants in the time of the COVID-19 pandemic are as follows. Both experience satisfaction and perceived enjoyment have strong direct impacts on experience extension. The impact of experience satisfaction on experience extension is moderated by both perceived risk reduction of viral infection and trust. The effect of perceived enjoyment on experience satisfaction is moderated by the novelty seeking tendency of customers.

However, it should be noted that the current study was constrained by several limitations. The limited mobility of the researchers, affected by Oman's COVID-19 restrictions, necessitated that the data collection be conducted using online means. As such, future researchers should recreate the present study by using physical methods of data collection instead. Moreover, the current study was essentially limited to Muscat city. As can be identified in the work of Tuomi et al. (2020), culture plays a crucial role in the way that people perceive robot service. Therefore, future researchers should replicate this study in various other locations to confirm the applicability of the results. Lastly, this study followed a cross sectional design, and there might, therefore, be issues concerning result bias, causality and incidence. As such, future researchers should investigate customer satisfaction with robot service restaurants should using longitudinal designs.

## Declarations

### Author contribution statement

Osman El-Said: Conceived and designed the experiments; Analyzed and interpreted the data; Contributed reagents, materials, analysis tools or data; Wrote the paper.

Sara Al Hajri: Conceived and designed the experiments; Performed the experiments; Analyzed and interpreted the data; Wrote the paper.

### Funding statement

The German University of Technology in Oman financed the Article Publishing Charges (APC).

### Data availability statement

Data will be made available on request.

### Declaration of interests statement

The authors declare no conflict of interest.

### Additional information

No additional information is available for this paper.
